# Elevated albumin corrected anion gap is associated with poor in-hospital prognosis in patients with cardiac arrest: A retrospective study based on MIMIC-IV database

**DOI:** 10.3389/fcvm.2023.1099003

**Published:** 2023-03-23

**Authors:** Beiping Hu, Lei Zhong, Meng Yuan, Jie Min, Lili Ye, Jianhong Lu, Xiaowei Ji

**Affiliations:** Department of Intensive Care Unit, Huzhou Central Hospital, the Affiliated Huzhou Hospital of Zhejiang University School of Medicine, Huzhou, China

**Keywords:** albumin corrected anion gap, cardiac arrest, intensive care unit, prognosis, MIMIC-IV database

## Abstract

**Background:**

Cardiac arrest(CA) is one of the most leading causes of death. Most of the indicators which used to predict the prognosis of patients with CA are not recognized. Previous studies have suggested that albumin corrected anion gap (ACAG) is associated with recovery of spontaneous circulation in patients with CA, but the predictive value of ACAG for prognosis has not been investigated. This study aims to explore the relationship between ACAG and prognosis during hospitalization in patients with CA.

**Methods:**

The baseline data of adult patients with CA hospitalized in the intensive care unit (ICU) from 2008 to 2019 in the American Intensive Care Database (MIMIC-IV, version v2.0) were collected. According to the in-hospital prognosis, patients were divided into survival and non-survival group. Based on the criteria of ACAG level in the previous literature, patients enrolled were divided into normal ACAG (12–20 mmol/L) and high ACAG (>20 mmol/L) group. The basic information of patients during hospitalization were compared and analyzed between the two groups with propensity score matching (PSM). The Kaplan-Meier method was used to compare the cumulative survival rates of normal ACAG and high ACAG groups before and after matching. Restricted cubic spline (RCS) method and multivariate COX proportional hazards regressions were used to analyze whether elevated ACAG was associated with all-cause mortality during hospitalization.

**Results:**

A total of 764 patients were included. A matched cohort (*n* = 310) was obtained after PSM analysis. The mortality rate before and after matching in the high ACAG group was higher than that in the normal ACAG group (*χ*^2^ = 25.798; *P* < 0.001; *χ*^2^ = 6.258; *P *= 0.012) The Kaplan-Meier survival analysis before and after matching showed that the cumulative survival rate of the high ACAG group was lower (*P* < 0.05). RCS analysis showed that ACAG had a non-linear relationship with the risk of in-hospital all-cause mortality (*χ*^2^ = 6.060, *P* < 0.001). Multivariate COX regression analysis before and after PSM suggested that elevated ACAG was an independent risk factor for all-cause mortality in patients with CA during hospitalization (*P *< 0.01).

**Conclusions:**

Elevated ACAG is associated with increased all-cause mortality in patients with CA during hospitalization, it can be an independent risk factor for poor prognosis in patients with CA and remind clinicians to pay more attention to these patients.

## Introduction

Cardiac arrest (CA) is one of the most concerned public health events all over world with high morbidity and mortality, and it is also one of the most leading causes of death. In the United States, more than 300,000 hospitalized patients experience cardiac arrest each year (1), and only 25 percent patients survive to hospital discharge (2). According to statistics, the global annual incidence rate is as high as 50–110/100,000 (3). Therefore, early and accurately determination of the prognosis is particularly important. Although there are many indicators used to predict the prognosis of patients with CA, such as lactate, neuron-specific enolase, end-tidal carbon dioxide, anion gap (AG), fibroblast growth factor 23, growth differentiation factor-15, etc., the diagnostic value of most indicators has not been confirmed, only the NSE has been recommended by the 2006 American Association of Neurologists guidelines (4–9). Thence, it is still very imperative to find simple but efficient indicators to predict the prognosis of patients with CA.

Acid-base disorders, including metabolic acidosis most commonly encountered in the intensive care unit (ICU), have been shown to be associated with morbidity and mortality (10). AG, which can help clinicians determine the type of acid-base disorders, especially for the metabolic acidosis refers to the difference between unmeasured cations and unmeasured anions in serum. However, ICU patients are often complicated by hypoalbuminemia, and albumin accounts for the majority of unmeasured anions in the body. Due to the charge of albumin, the results will be falsely negative, masking the increased AG level and leading to misjudgment (11). Therefore, scholars insisted that albumin corrected anion gap (ACAG) is more appropriate for the diagnosis of metabolic acidosis (12).

ACAG has been reported to be associated with outcomes in patients with sepsis (13) and kidney disease (14). Elevated ACAG (>21.25 mmol/L) can increase the mortality of patients with sepsis in ICU (15). Hagiwara et al. have already explored the association between ACAG and recovery of spontaneous circulation (ROSC) in patients with CA (16), but the predictive value of ACAG on prognosis of patients with CA has not been further investigated. Therefore, the study intends to explore the correlation between ACAG and all-cause mortality of patients with CA during hospitalization.

## Materials and methods

### Sources of data

The data we analyzed were extracted from MIMIC-IV (Medical Information Mart for Intensive Care IV, v2.0) database (17), a large and publicly available critical care database approved by the Institutional Review Boards of Beth Israel Deaconess Medical Center and the Massachusetts Institute of Technology. The database includes 76,540 ICU patients admitted to Beth Israel Deaconess Medical Center in Boston from 2008 to 2019. One author of our group was approved to access the database after accomplishing the Collaborative Institutional Training Initiative (CITI) program course (Record ID: 36142713).

### Study population

ICU patients diagnosed with CA from 2008 to 2019 in the MIMIC database were enrolled in the study. Only adult patients (order than 18 years old) first admitted to the ICU were included. Exclusion criteria are as follows: (1) ICU length of stay < 24 h; (2) Missing key data such as ACAG.

### Data extraction

Data were acquired from the MIMIC-IV database using the structured query language with PostgreSQL 10.13. Clinical data on age, sex, SOFA score, and comorbidities of the study population were collected. At the same time, laboratory test results including serum AG, albumin, ACAG, bicarbonate, white blood cell (WBC) count, hemoglobin, red blood cell distribution width (RDW), platelet, aspartate aminotransferase (AST), blood urea nitrogen (BUN), serum creatinine, the international normalized ratio (INR), blood glucose, total serum calcium, serum chlorine were recorded at the first test after admission to the ICU.

### Groups and primary endpoints

Based on the previously reported literature (18), ACAG was calculated as: ACAG (mmol/L) = AG + [44—albumin(g/L)] × 0.25. According to the prognosis during hospitalization, the included patients were divided into survival group (*n* = 391) and non-survival group (*n* = 373). Then, referring to previous study (15), subjects were divided into normal ACAG group (12–20 mmol/L, *n* = 375) and high ACAG group (>20 mmol/L, *n* = 389).

The endpoint of the study was all-cause mortality during hospitalization.

### Statistical analysis

Continuous variables conformed to normal distribution, were expressed as mean ± standard deviation (`× ± s), with the analysis of t-test method; if variables did not conformed to normal distribution, they were expressed as median (interquartile range) [M (QL, QU)], with the analysis of nonparametric (Mann-Whitney U) test between the two groups. Categorical data were presented as constituent ratios and analyzed by the method of chi-square test.

Propensity score matching (PSM) analysis which was conducted using a 1:1 nearest neighbor matching algorithm with a caliper of 0.3 was performed to reduce bias between the survival and non-survival groups. Also, PSM analysis between normal ACAG and high ACAG was performed. Kaplan-Meier curves were drawn before and after matching, and the cumulative survival rate during hospitalization was compared between the the normal and high ACAG groups by the long-rank test.

Restricted cubic spline (RCS) was utilized to analyze the relevance between ACAG at ICU admission and risk of all-cause mortality during hospitalization.

Variables with a *P* value less than 0.10 in univariate analysis between survival and non-survival groups were included in multivariate Cox regression analysis, and the results were expressed as hazard ratio (HR) with 95% confidence interval (CI).

Data analysis was performed using Stata 14.0 software and R programming language version 4.2.0. Statistical significance was defined as a two-tailed *P*-value less than 0.05.

## Resutls

### Subject characteristics

**Figure 1:**
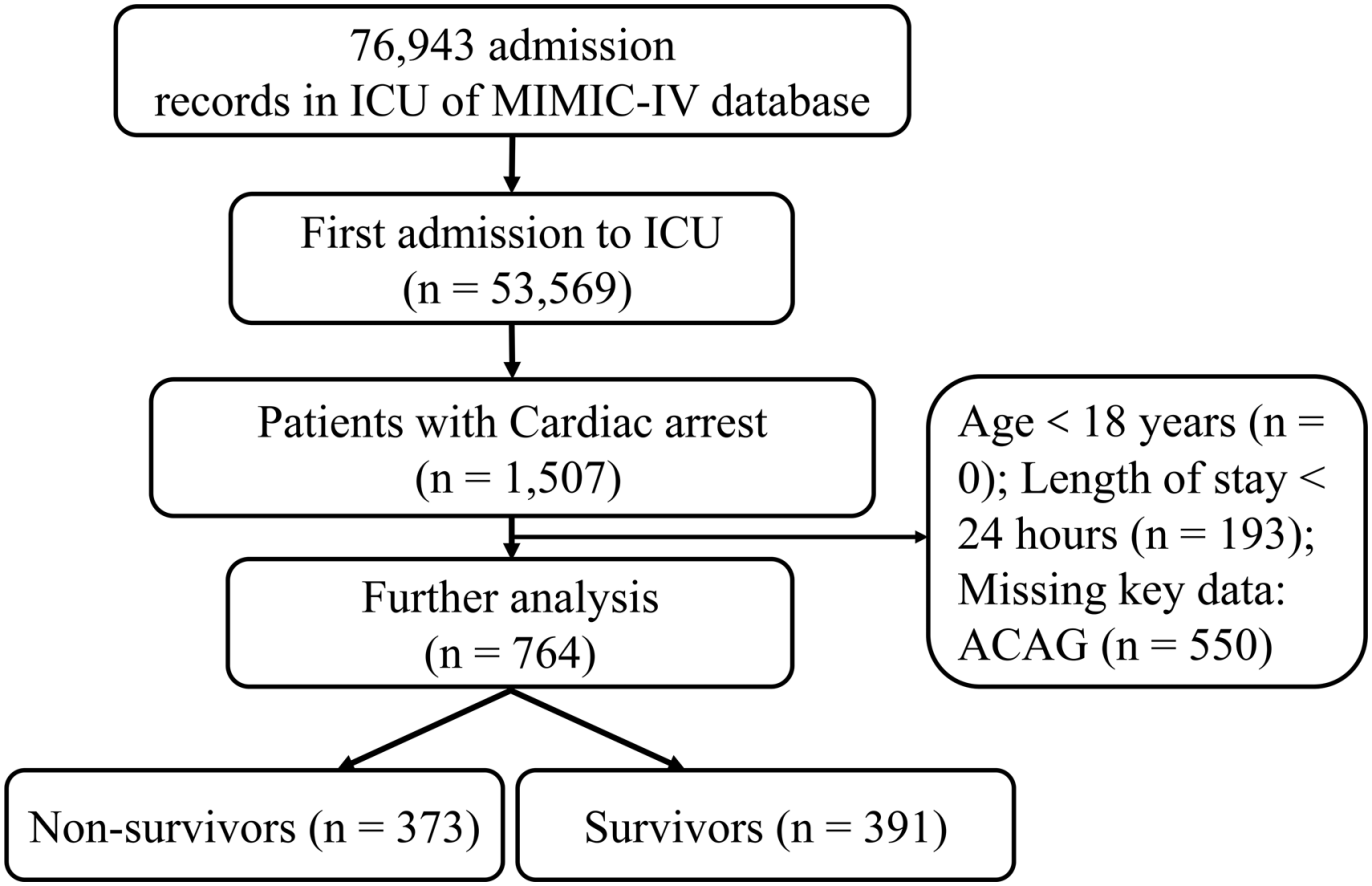
The flowchart of patients screening.

We retrieved the data of 764 eligible patients from the MIMIC-IV database ultimately, as shown in the flowchart in Figure 1. The average age of the participants was 64.71 ± 16.81 years. 310 patients were matched after PSM. Before PSM, compared with the survival group, the SOFA score, AG, ACAG, RDW, AST, BUN, creatinine, and INR values were higher in the non-survival group, as was the incidence of chronic obstructive pulmonary disease (COPD). However, the HCO_3_-, albumin, hemoglobin values and the incidence of VAP in the survival group were significantly higher than those in the non-survival group, and the length of hospital stay in the survival group was also shorter (all *P *< 0.05). After PSM, the results showed that, compared with the survival group, ACAG, RDW, BUN and INR in the non-survival group were still higher, albumin, the incidence of VAP and CKD was lower, and the hospital stay in the non-survival group was longer (*P* < 0.05), as shown in Table 1. Characteristics of the participants between the normal and high ACAG groups was also presented in Table 2. After PSM, characteristics of normal and high ACAG groups were more comparable between the two groups.

**Table 1:** Characteristics of the study population between survival and non-survival groups.

Variables	Before PSM				After PSM			
	Survival group (*n* = 391)	Non-survival group (*n* = 373)	*t/Z/χ^2^*	*P-value*	Survival group (*n* = 148)	Non-survival group (*n* = 162)	*t/Z/χ^2^*	*P-value*
Age (year)	63.64 ± 16.68	65.84 ± 16.90	−1.813	0.070	64.39 ± 16.26	66.90 ± 17.12	−1.322	0.187
Sex, male, *n* (%)	254 (64.96)	227 (60.86)	1.379	0.240	101 (68.24)	91 (56.17)	4.780	0.029
SOFA score	8.92 ± 4.42	10.19 ± 4.39	−3.991	<0.001	9.32 ± 4.04	10.02 ± 9.42	−1.561	0.120
AG (mmol/L)	16.86 ± 4.81	18.42 ± 5.58	−4.122	<0.001	16.47 ± 3.68	17.25 ± 4.00	−1.783	0.076
Albumin (g/L)	30.95 ± 6.37	28.54 ± 6.92	5.005	<0.001	30.83 ± 6.49	28.80 ± 7.10	2.617	0.009
ACAG (mmol/L)	20.13 ± 5.09	22.28 ± 5.74	−5.492	<0.001	19.77 ± 3.58	21.05 ± 3.78	−3.071	0.002
HCO3- (mmol/L)	21.04 ± 5.03	19.88 ± 5.06	3.192	0.002	20.68 ± 3.73	20.78 ± 3.85	−0.235	0.814
WBC (×10^9^/L)	13.00 (8.60, 17.80)	13.30 (9.30, 19.40)	−1.427	0.154	13.70 (9.05, 19.90)	14.1 (8.70, 18.90)	0.474	0.635
Hemoglobin (g/L)	115.86 ± 27.13	111.43 ± 27.75	2.234	0.026	116.68 ± 26.63	113.31 ± 27.96	1.081	0.280
RDW (%)	14.81 ± 2.30	15.43 ± 2.30	−3.737	<0.001	14.71 ± 2.26	15.31 ± 2.25	−2.320	0.021
Platelet (×10^9^/L)	197.00 (145.00, 249.00)	198.00 (142.00, 274.00)	−0.751	0.453	204.00 (150.50, 261.50)	190.50 (141.00, 269.00)	1.136	0.256
AST (U/L)	92.00 (41.00, 220.00)	119.00 (46.00, 348.00)	−2.746	0.006	89.50 (39.50, 232.00)	107.50 (43.00, 314.00)	−1.378	0.168
BUN (mg/dl)	22.00 (15.00, 34.00)	26.00 (18.00, 44.00)	−4.018	<0.001	23.00 (15.00, 35.00)	25.00 (19.00, 38.00)	−2.137	0.033
Creatinine (umol/L)	106.08 (70.72, 167.96)	123.76 (79.56, 185.64)	−2.568	0.010	106.08 (79.56, 163.54)	106.08 (88.4, 159.12)	−0.543	0.587
INR	1.30 (1.10, 1.50)	1.40 (1.20, 1.80)	−5.097	<0.001	1.20 (1.10, 1.50)	1.30 (1.20, 1.70)	−3.744	<0.001
Glucose (mmol/L)	8.72 (6.83, 12.39)	9.56 (7.00, 13.39)	−1.401	0.161	9.47 (6.97, 13.61)	9.83 (7.83, 13.83)	−0.524	0.600
Total serum calcium (mmol/L)	2.07 ± 0.30	2.05 ± 0.33	1.088	0.277	2.03 ± 0.27	2.04 ± 0.26	−0.546	0.586
**Comorbidities, *n* (%)**
Hypertension	153 (39.13)	159 (42.63)	0.966	0.326	57 (38.51)	76 (46.91)	2.228	0.136
Diabetes	132 (33.76)	131 (35.12)	0.157	0.692	45 (30.41)	58 (35.80)	1.015	0.314
Cardiogenic shock	83 (21.23)	73 (19.57)	0.322	0.570	35 (23.65)	33 (20.37)	0.485	0.486
AMI	74 (18.93)	60 (16.09)	1.065	0.302	34 (22.97)	30 (18.52)	0.937	0.333
AKI	331 (84.65)	331 (88.74)	2.754	0.097	131 (88.51)	149 (91.98)	1.060	0.303
VAP	57 (14.58)	35 (9.38)	4.863	0.027	22 (14.86)	11 (6.79)	5.302	0.021
COPD	40 (10.23)	56 (15.01)	3.975	0.046	17 (11.49)	25 (15.43)	1.028	0.311
CKD	100 (25.58)	84 (22.52)	0.975	0.324	41 (27.70)	29 (17.90)	4.250	0.039
Length of hospital stay (day)	14.92 (8.42,26.13)	6.25 (2.75,13.04)	11.772	<0.001	15.79 (7.83, 27.02)	6.73 (2.75, 13.04)	7.200	<0.001

SOFA, sequential organ failure assessment; AG, anion gap; ACAG, albumin corrected anion gap; HCO3-, bicarbonate; WBC, white blood cell; RDW, red blood cell distribution width; AST, aspartate aminotransferase; BUN, blood urea nitrogen; INR, international normalized ratio; AMI, acute myocardial infarction; AKI, acute kidney injury; VAP, ventilator associated pneumonia; COPD, chronic obstructive pulmonary disease; CKD, chronic kidney disease; PSM propensity score matching.

**Table 2:** Characteristics of the study population between normal and high ACAG groups before and after PSM.

Variables	Before PSM				After PSM			
	Normal ACAG group (*n* = 375)	High ACAG group (*n* = 389)	*t/Z/χ^2^*	*P-value*	Normal ACAG group (*n* = 155)	High ACAG group (*n* = 155)	*t/Z/χ^2^*	*P-value*
Age (year)	65.36 ± 16.79	64.08 ± 16.83	1.050	0.294	65.97 ± 17.08	65.44 ± 16.43	0.277	0.782
Sex, male, *n* (%)	244 (65.07)	237 (60.93)	1.404	0.236	95 (61.29)	97 (62.58)	0.055	0.815
SOFA score	7.99 ± 4.32	11.04 ± 4.04	−10.078	<0.001	9.70 ± 4.09	9.66 ± 3.83	0.086	0.936
AG (mmol/L)	13.81 ± 2.33	21.30 ± 4.63	−28.092	<0.001	14.21 ± 2.39	19.55 ± 3.13	−16.890	<0.001
Albumin (g/L)	31.49 ± 6.31	28.13 ± 6.76	7.103	<0.001	30.70 ± 6.80	28.85 ± 6.86	2.387	0.018
ACAG (mmol/L)	16.94 ± 2.09	25.27 ± 4.64	−31.795	<0.001	17.53 ± 1.95	23.34 ± 2.69	−21.805	<0.001
HCO3- (mmol/L)	22.94 ± 4.01	18.09 ± 4.86	15.001	<0.001	20.71 ± 3.59	20.76 ± 3.99	−0.120	0.905
WBC (×10^9^/L)	11.8 (8.60, 16.70)	14.40 (9.10, 20.70)	−4.193	<0.001	13.40 (9.30, 18.8)	14.40 (8.60, 19.50)	−0.424	0.672
Hemoglobin (g/L)	115.37 ± 25.49	112.09 ± 29.25	1.651	0.099	113.92 ± 27.14	115.92 ± 27.59	−0.646	0.519
RDW (%)	14.87 ± 2.31	15.35 ± 2.31	−2.859	0.004	15.02 ± 2.34	15.03 ± 2.20	−3.073	0.942
Platelet (×10^9^/L)	198.00 (150.00, 256.00)	198.00 (138.00, 264.00)	0.485	0.628	190.00 (142.00, 261.00)	206.00 (147.00, 274.00)	−1.287	0.198
AST (U/L)	72.00 (36.00, 149.00)	153.00 (60.00, 418.00)	−7.843	<0.001	84.00 (39.00, 231.00)	116.00 (47.00, 275.00)	−1.950	0.051
BUN (mg/dl)	20.00 (14.00, 29.00)	29.00 (19.00, 51.00)	−8.241	<0.001	25.00 (18.00, 35.00)	23.00 (18.00, 38.00)	0.410	0.682
Creatinine (umol/L)	188.40 (70.72, 123.76)	150.28 (97.24, 221.00)	−11.183	<0.001	106.08 (79.56, 159.12)	114.92 (88.40, 159.12)	−1.014	0.311
INR	1.30 (1.10, 1.50)	1.80 (1.20, 1.80)	−4.815	<0.001	1.30 (1.10, 1.60)	1.30 (1.10, 1.70)	−0.935	0.350
Glucose (mmol/L)	8.44 (6.67, 11.00)	10.22 (7.28, 14.72)	−5.510	<0.001	9.28 (6.83, 12.39)	10.28 (7.83, 14.56)	−1.857	0.063
Total serum calcium (mmol/L)	2.06 ± 0.26	2.06 ± 0.36	−0.072	0.943	2.05 ± 0.29	2.03 ± 0.24	0.604	0.547
**Comorbidities, *n* (%)**
Hypertension	172 (45.87)	140 (35.99)	7.710	0.005	68 (43.87)	65 (62.58)	0.119	0.731
Diabetes	103 (27.47)	160 (41.13)	15.793	<0.001	51 (32.90)	52 (33.55)	0.015	0.904
Cardiogenic shock	61 (16.37)	95 (24.42)	7.814	0.005	33 (21.29)	35 (22.58)	0.075	0.784
AMI	61 (16.27)	73 (18.77)	0.825	0.364	28 (18.06)	36 (23.23)	1.260	0.262
AKI	303 (80.80)	359 (92.29)	21.780	<0.001	141 (90.97)	139 (89.68)	0.148	0.701
VAP	44 (11.73)	48 (12.34)	0.066	0.797	17 (10.97)	16 (10.32)	0.034	0.854
COPD	55 (14.67)	41 (10.54)	2.960	0.085	20 (12.90)	22 (14.19)	0.110	0.740
CKD	62 (16.53)	122 (31.36)	22.965	<0.001	34 (21.94)	36 (23.23)	0.074	0.786
Length of hospital stay (day)	10.75 (5.42, 21.92)	9.58 (4.08,18.79)	2.396	0.017	10.83 (6.21, 24.33)	8.58 (4.79, 16.83)	2.350	0.019

SOFA, sequential organ failure assessment; AG, anion gap; ACAG, albumin corrected anion gap; HCO3-, bicarbonate; WBC, white blood cell; RDW, red blood cell distribution width; AST, aspartate aminotransferase; BUN, blood urea nitrogen; INR, international normalized ratio; AMI, acute myocardial infarction; AKI, acute kidney injury; VAP, ventilator associated pneumonia; COPD, chronic obstructive pulmonary disease; CKD, chronic kidney disease; PSM propensity score matching.

### All-cause mortality of the two groups

The all-cause mortality rate of the included patients during hospitalization was 48.82%. The mortality rate in the high ACAG group (57.84%) was significantly higher than that in the normal ACAG group (39.47%, *χ*^2^ = 25.798; *P* < 0.001). After PSM, the mortality rate in the high ACAG group (59.35%) was also higher (*χ*^2^ = 6.258; *P = *0.012), as shown in Table 3.

**Table 3:** All-cause mortality during hospitalization between two groups.

Before PSM					After PSM			
Group	Survivors (*n* = 391)	Non-survivors (*n* = 373)	χ^2^	*P*	Survivors (*n* = 148)	Non-survivors (*n* = 162)	χ^2^	*P*
Normal ACAG	227 (60.53)	148 (39.47)	25.798	<0.001	85 (54.83)	70 (45.16)	6.258	0.012
High ACAG	164 (42.16)	225 (57.84)			63 (40.65)	92 (59.35)		

ACAG, albumin corrected anion gap; PSM, propensity score matching.

### Kaplan-Meier survival curve analysis

**Figure 2:**
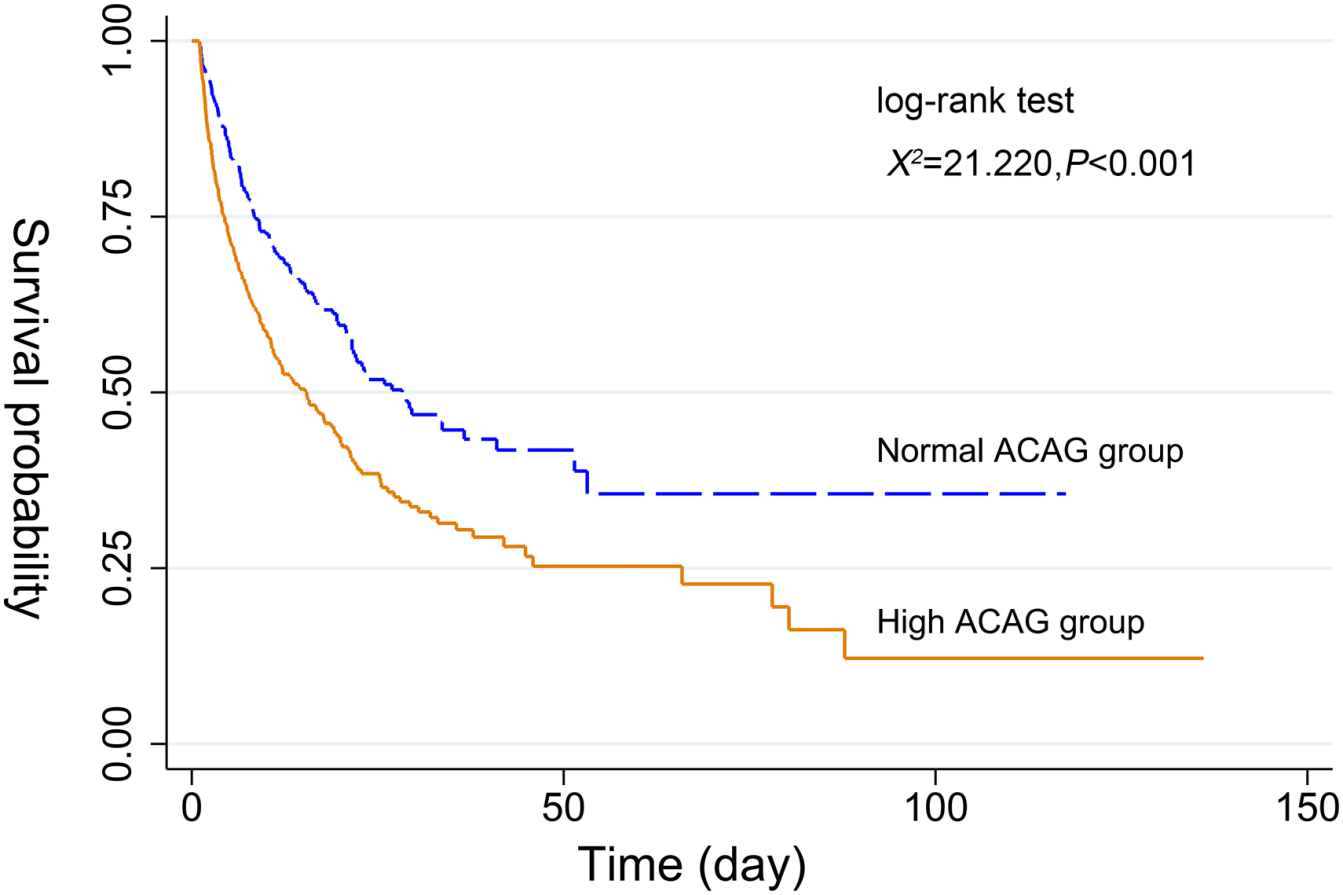
Kaplan-Meier survival curve of cumulative survival rate during hospitalization for the normal and high ACAG groups before PSM.

**Figure 3:**
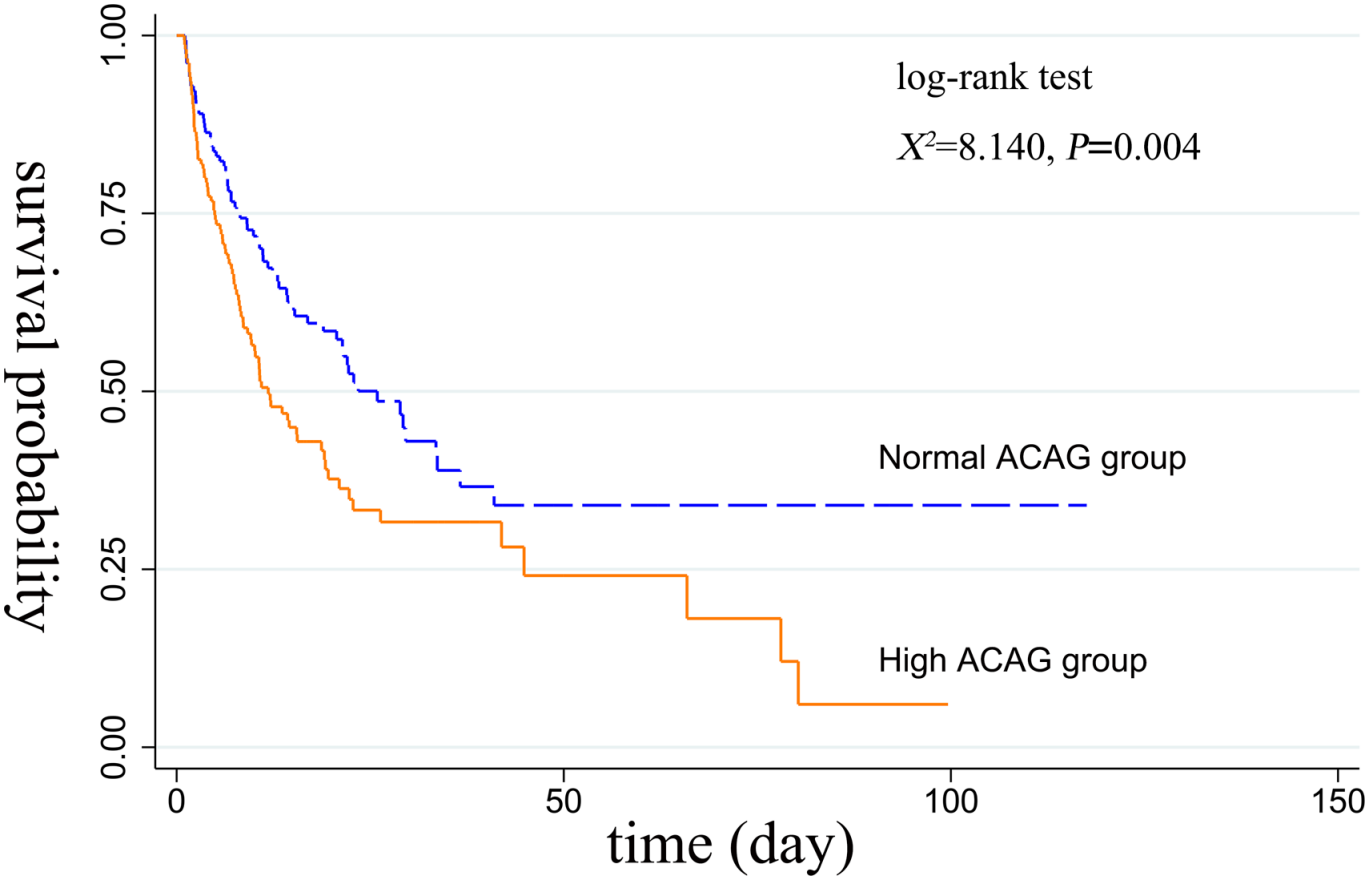
Kaplan-Meier survival curve of cumulative survival rate during hospitalization for the normal and high ACAG groups after PSM.

Before or after matching, the Kaplan-Meier survival curves in Figures 2, 3 showed that compared with the normal ACAG group, the cumulative survival rate of patients with CA was significantly lower (log-rank test, *χ*^2 ^= 21.220, *P* < 0.001; *χ*^2^ = 8.140, *P = *0.004) in the high ACAG group during hospitalization.

### Correlation between ACAG and risk of all-cause mortality during hospitalization in patients with ca

RCS showed that there was a non-linear relationship between ACAG at ICU admission and the risk of all-cause mortality during hospitalization in patients with CA (*χ*^2 ^= 6.060, *P *= 0.048). When ACAG was 20.27 mmol/L, its HR was 1.

**Figure 4:**
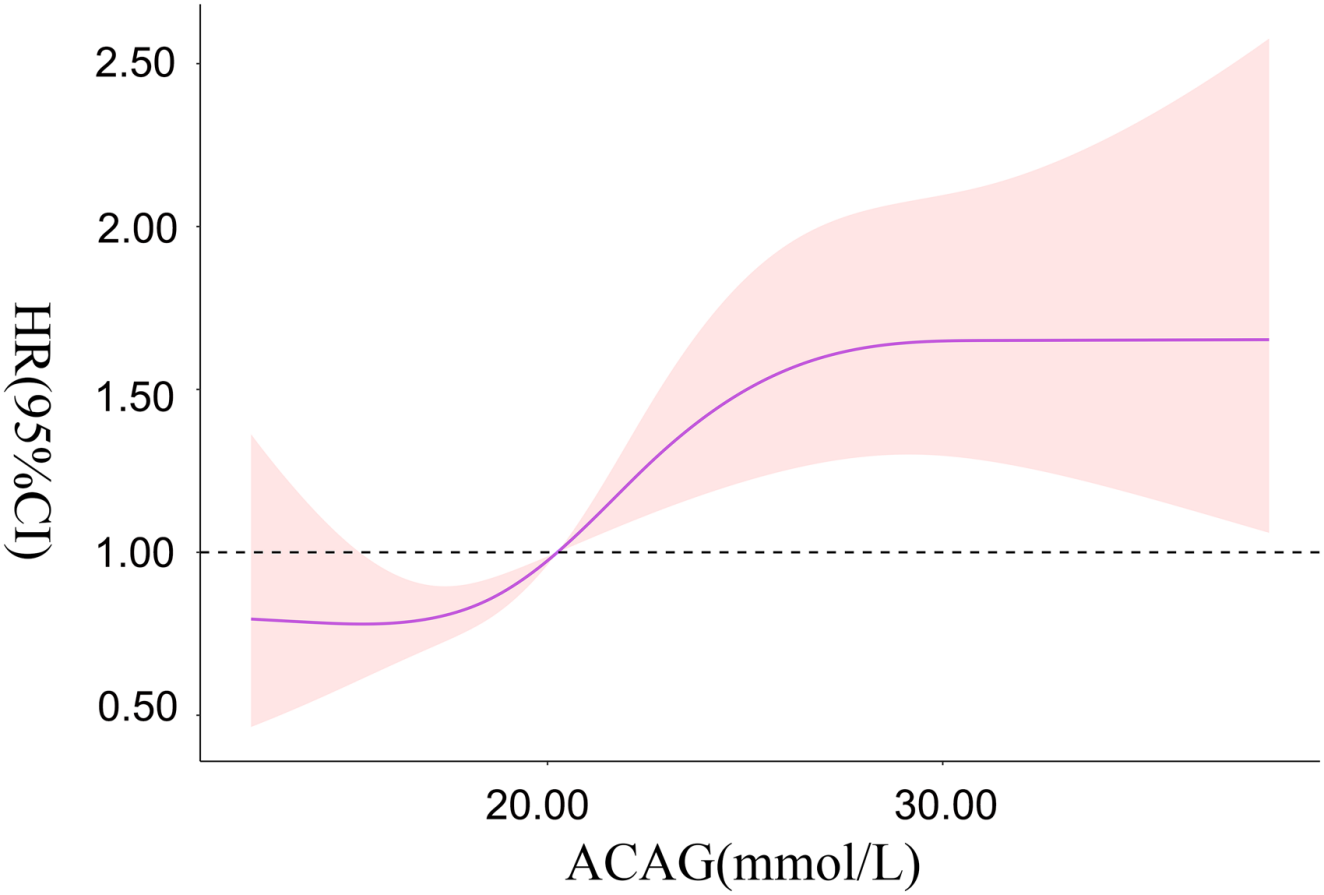
The correlation between ACAG and the risk of all-cause mortality in patients with CA.

Overall, with the increase of ACAG, the risk of all-cause mortality during hospitalization in patients with CA increased accordingly. When ACAG was >30.00 mmol/L, the risk of all-cause mortality was always at a high level and was relatively stable, as shown in Figure 4.

### Elevated ACAG was an independent risk factor for increased all-cause mortality

It could be concluded from Table 4 that in Model I compared to the normal ACAG group, the HR (95% CI) of all-cause mortality during hospitalization for the high ACAG group was 1.621 (1.317–1.995), indicating that elevated ACAG (>20 mmol/L) was a predictor of increased all-cause mortality during hospitalization in patients with CA. While, after adjusting for multiple confounding factors, including age, SOFA score, Bicarbonate, hemoglobin, RDW, AST, BUN, creatinine, INR and the incidence of AKI, VAP and COPD, the Cox proportional hazard analysis showed that the HR (95%CI) was 1.559 (1.217–1.998), it was concluded that the elevated ACAG (>20 mmol/L) was an independent risk factor of increased all-cause mortality during hospitalization in patients with CA.

**Table 4:** Cox proportional hazard analysis of all-cause mortality in patients with CA during hospitalization.

Variable		Model I			Model II		
		HR	95% CI	*P*-value	HR	95% CI	*P*-value
Before PSM	Normal ACAG	1			1
	High ACAG	1.621	1.317–1.995	<0.001	1.559	1.217–1.998	<0.001
After PSM	Normal ACAG	1			1		
	High ACAG	1.569	1.148–2.143	0.005	1.576	1.151–2.157	0.005

Before PSM: Model I: No covariates were adjusted.

Model II: adjusted for age, SOFA score, Bicarbonate, hemoglobin, RDW, AST, BUN, creatinine, INR, the incidence of VAP, AKI and COPD.

After PSM: Model I: No covariates were adjusted.

Model II: adjusted for RDW, BUN, INR, sex and the incidence of VAP and CKD.

Abbreviations: HR, hazard ratio, 95% CI 95% confidence interval; CA, cardiac arrest; ACAG, albumin corrected anion gap; SOFA, sequential organ failure assessment; RWD, red blood cell distribution width; AST, aspartate aminotransferase; BUN, blood urea nitrogen; INR, international normalized ratio; VAP, ventilator associated pneumonia; AKI, acute kidney injury; CKD, chronic kidney disease; COPD, chronic obstructive pulmonary disease; PSM, propensity score matching.

After PSM, whether in model I or model II, the results still suggested that elevated ACAG was an independent risk factor for all-cause death during hospitalization in patient with CA (*P* < 0.01), as shown in Table 4.

## Discussion

The current study found that ACAG in the survival group was lower than that in the non-survival group, and compared to the normal ACAG group, all-cause mortality in the high ACAG group was higher. The Kaplan-Meier survival curve showed that the cumulative survival rate during hospitalization in the high ACAG group was significantly lower. RCS showed a corresponding increase in the risk of all-cause mortality with the increasing ACAG. When ACAG was >30.00 mmol/L, the risk of all-cause mortality was always at a high level. Further analysis of multivariate Cox proportional hazards regression showed that elevated ACAG (>20 mmol/L) was an independent risk factor of increased all-cause mortality during hospitalization in patients with CA. Therefore, when ACAG at ICU admission is greater than 20 mmo/L, the prognosis of the patients during hospitalization can be preliminary judged, and more attention should be paid to such patients.

Homeostasis is the state of steady conditions to maintain normal life activities, while acid-base balance lays the foundation for the body to perform various functions normally. Because of the imbalance in the production of acids or bases, the acid-base homeostasis can be disrupted, resulting in acid-base balance disorders, the most common of which is metabolic acidosis. It can be life-threatening if it is not controlled in time or treated effectively. Metabolic acidosis is a serious electrolyte disorder with a decrease in bicarbonate concentration. It can be divided into high AG normochloremic and normal AG hyperchloremic metabolic acidosis (19). Recently, as a new serological marker, AG has attracted the attention of scholars because of its easy availability, and provided important clues for the diagnosis or prognosis of various diseases. Studies had shown that elevated AG is associated with poor prognosis, and was an independent predictor for predicting in-hospital mortality in patients with CA (5). Zhang et al. found that serum AG value ≥ 11.15 mmol/L was associated with cardiovascular events during 1-year follow-up in patients with acute coronary syndrome (20). Meanwhile, AG was an independent risk factor for predicting in-hospital mortality in patients with acute ischemic stroke (21). In addition, several research showed that high AG was also correlated with increased risks of cardiovascular or death events in disease such as renal diseases (22), sepsis (23), acute pancreatitis (24), disseminated intravascular coagulation (25), aortic aneurysm (26) and so on.

However, ICU patients are mostly accompanied by hypoalbuminemia. Because of the charge of albumin, AG may appear falsely “normal”, which affects the physicians’ judgment on the accuracy of the results. ACAG is albumin-corrected AG, which can relatively improve the diagnostic sensitivity of metabolic acidosis and the predictive value for prognosis in ICU patients. Therefore, some scholars have proposed that ACAG is a more suitable tool for diagnosis of disease and prediction of prognosis in ICU patients (12). At present, many scholars have initiated to explore the relationship between ACAG and outcomes of patients with different diseases. Hu et al. found that ACAG (>21.25 mmol/L) could predict the risk of in-hospital mortality in ICU patients with sepsis, and the predictive value of ACAG was superior to AG and albumin (13).The current evidence suggested that elevated ACAG (>20 mmol/L) at the initiation of continuous renal replacement therapy (CRRT) was associated with ICU all-cause mortality in AKI patients who underwent CRRT, and ACAG can serve as an early indicator of adverse outcomes for these patients (14). In 2013, a study by Hagiwara et al. had concluded that ACAG was more accurate in predicting ROSC in patients with CA. The next year, the team found that when the median ACAG was greater than 40.9 mmol/L, patients with CA were less likely to restore spontaneous circulation. However, the correlation between ACAG and the outcomes of patients with CA has never been investigated. We explored the correlation between ACAG and in-hospital all-cause mortality in patients with CA, and found that high ACAG was an independent risk factor for poor prognosis in those patients. Base on the studies above, clinicians should be particularly vigilant in patients with higher ACAG.

At present, SOFA score, RDW distribution, BUN, and Creatinine are significantly different between survivors and non-survivors which are established markers of severity. However, there are few studies in patients with cardiac arrest. Many scholars are still committed to looking for predictors of disease prognosis, so that the prognosis of patients can be judged in many ways. ACAG is a new inflammatory index, which has been found to be related to the prognosis of many diseases, such as AKI, sepsis and so on (13, 14). Our research results have complemented the previous research on ACAG and found that high ACAG was an independent risk factor for poor prognosis in patients with CA. At the same time, ACAG can be a new indicator that can provide new ideas for clinical practice, so that doctors can judge the prognosis of patients more accurately.

The strengths of our study are as follows. Firstly, the study is the first to explore the relationship between ACAG and in-hospital prognosis in patients with CA. Secondly, the data we extract from MIMIC-IV database comes from the real world, which is more convincing.

Nevertheless, there exist some shortcomings. First, this study is a retrospective study. second, we only tested ACAG on ICU admission without dynamically monitoring ACAG, which may change over time or condition of the patients during hospitalization. Furthermore, we did not group patients according to some confounding factors such as comorbidities and lacked subgroup analysis and sensitivity analysis. Therefore, further studies on the association between the dynamic changes of ACAG and the mortality of these patients are still needed to confirm our point.

## Conclusion

In conclusion, elevated ACAG (>20 mmol/L) was associated with higher in-hospital all-cause mortality in patients with CA. It can be an independent risk factor for poor prognosis in patients with CA. However, the conclusions still need to be further confirmed by prospective large-sample studies.

## Data Availability

The datasets presented in this study can be found in online repositories. The names of the repository/repositories and accession number(s) can be found in the article/Supplementary Material.

## References

[1] Holmberg MJ Ross CE Fitzmaurice GM Chan PS Duval-Arnould J Grossestreuer AV Annual incidence of adult and pediatric in-hospital cardiac arrest in the United States. Circ Cardiovasc Qual Outcomes. (2019) 12:e005580. 10.1161/CIRCOUTCOMES.119.005580 31545574 PMC6758564

[2] Andersen LW Holmberg MJ Berg KM Donnino MW Granfeldt A. In-hospital cardiac arrest: a review. Jama. (2019) 321:1200–10. 10.1001/jama.2019.1696 30912843 PMC6482460

[3] Efendijev I Nurmi J Castren M Skrifvars MB. Incidence and outcome from adult cardiac arrest occurring in the intensive care unit: a systematic review of the literature. Resuscitation. (2014) 85:472–9. 10.1016/j.resuscitation.2013.12.027 24412160

[4] Wijdicks EF Hijdra A Young GB Bassetti CL Wiebe S. Quality standards subcommittee of the American academy of N. Practice parameter: prediction of outcome in comatose survivors after cardiopulmonary resuscitation (an evidence-based review): report of the quality standards subcommittee of the American academy of neurology. Neurology. (2006) 67:203–10. 10.1212/01.wnl.0000227183.21314.cd 16864809

[5] Chen J Dai C Yang Y Wang Y Zeng R Li B The association between anion gap and in-hospital mortality of post-cardiac arrest patients: a retrospective study. Sci Rep. (2022) 12:7405. 10.1038/s41598-022-11081-3 35524151 PMC9076652

[6] Poppe M Stratil P Clodi C Schriefl C Nurnberger A Magnet I Initial end-tidal carbon dioxide as a predictive factor for return of spontaneous circulation in nonshockable out-of-hospital cardiac arrest patients: a retrospective observational study. Eur J Anaesthesiol. (2019) 36:524–30. 10.1097/EJA.0000000000000999 31742569

[7] Richter B Uray T Krychtiuk KA Schriefl C Lenz M Nurnberger A Growth differentiation factor-15 predicts poor survival after cardiac arrest. Resuscitation. (2019) 143:22–8. 10.1016/j.resuscitation.2019.07.028 31394153

[8] Spaich S Zelniker T Endres P Stiepak J Uhlmann L Bekeredjian R Fibroblast growth factor 23 (FGF-23) is an early predictor of mortality in patients with cardiac arrest. Resuscitation. (2016) 98:91–6. 10.1016/j.resuscitation.2015.11.012 26655587

[9] Zampieri FG Park M Ranzani OT Maciel AT de Souza HP da Cruz Neto LM Anion gap corrected for albumin, phosphate and lactate is a good predictor of strong ion gap in critically ill patients: a nested cohort study. Rev Bras Ter Intensiva. (2013) 25:205–11. 10.5935/0103-507X.20130036 24213083 PMC4031845

[10] Schricker S Schanz M Alscher MD Kimmel M. Metabolic acidosis: diagnosis and treatment. Med Klin Intensivmed Notfmed. (2020) 115:275–80. 10.1007/s00063-019-0538-y 30725274

[11] Nanji AA Campbell DJ Pudek MR. Decreased anion gap associated with hypoalbuminemia and polyclonal gammopathy. Jama. (1981) 246:859–60. 10.1001/jama.1981.03320080045027 6166764

[12] Hatherill M Waggie Z Purves L Reynolds L Argent A. Correction of the anion gap for albumin in order to detect occult tissue anions in shock. Arch Dis Child. (2002) 87:526–9. 10.1136/adc.87.6.526 12456555 PMC1755806

[13] Hu T Zhang Z Jiang Y. Albumin corrected anion gap for predicting in-hospital mortality among intensive care patients with sepsis: a retrospective propensity score matching analysis. Clin Chim Acta. (2021) 521:272–7. 10.1016/j.cca.2021.07.021 34303712

[14] Zhong L Xie B Ji XW Yang XH. The association between albumin corrected anion gap and ICU mortality in acute kidney injury patients requiring continuous renal replacement therapy. Intern Emerg Med. (2022) 17:2315–22. 10.1007/s11739-022-03093-8 PMC9652260 36112320

[15] He X Liao X Xie Z Jiang C Kang Y. Albumin corrected anion gap is an independent risk factor for long-term mortality of patients with sepsis. Zhonghua Wei Zhong Bing Ji Jiu Yi Xue. (2017) 29:117–21. 10.3760/cma.j.issn.2095-4352.2017.02.005 28625257

[16] Hagiwara S Oshima K Furukawa K Nakamura T Ohyama Y Tamura J. The significance of albumin corrected anion gap in patients with cardiopulmonary arrest. Ann Thorac Cardiovasc Surg. (2013) 19:283–8. 10.5761/atcs.oa.12.01942 23232266

[17] Goldberger AL Amaral LA Glass L Hausdorff JM Ivanov PC Mark RG Physiobank, PhysioToolkit, and PhysioNet: components of a new research resource for complex physiologic signals. Circulation. (2000) 101:E215–220. 10.1161/01.cir.101.23.e215 10851218

[18] Lopez A Garcia B Gomez A Gonzalez L Gonzalez N Martin L Concordance of the ions and GAP anion obtained by gasometry vs standard laboratory in critical care. Med Intensiva. (2019) 43:521–7. 10.1016/j.medin.2018.06.009 30193741

[19] Tanemoto M. Progression of metabolic acidosis in chronic kidney disease. Kidney Dis. (2020) 6:59–63. 10.1159/000502380 PMC6995968 32021875

[20] Zhang Q Cheng C Zhang Q Cheng C Xu R Wu C. Predictive value of neutrophil-to-lymphocyte ratio on microcirculatory disturbance in ST-segment elevation myocardial infarction patients undergoing primary percutaneous coronary intervention. Chin J Crit Care Med (Electronic Edition). (2020) 13:351–5. 10.3877/cma.j.issn.1674-6880.2020.05.006

[21] Jhou HJ Chen PH Yang LY Chang SH Lee CH. Plasma anion gap and risk of in-hospital mortality in patients with acute ischemic stroke: analysis from the MIMIC-IV database. J Pers Med. (2021) 11:1–10. 10.3390/jpm11101004 34683145 PMC8541378

[22] Asahina Y Sakaguchi Y Kajimoto S Hattori K Doi Y Oka T Time-updated anion gap and cardiovascular events in advanced chronic kidney disease: a cohort study. Clin Kidney J. (2022) 15:929–36. 10.1093/ckj/sfab277 35498899 PMC9050520

[23] Zhu Y He Z Jin Y Zhu S Xu W Li B Serum anion gap level predicts all-cause mortality in septic patients: a retrospective study based on the MIMIC III database. J Intensive Care Med. (2022) 38:349–57. 10.1177/08850666221123483 36066040

[24] Gong F Zhou Q Gui C Huang S Qin Z. The relationship between the serum anion gap and all-cause mortality in acute pancreatitis: an analysis of the MIMIC-III database. Int J Gen Med. (2021) 14:531–8. 10.2147/IJGM.S293340 33642873 PMC7903165

[25] Hu B Cao J Hu Y Qin Z Wang J. The association between serum anion gap and all-cause mortality in disseminated intravascular coagulation patients: a retrospective analysis. Int J Gen Med. (2021) 14:4535–44. 10.2147/IJGM.S318334 34429638 PMC8379146

[26] Chen Q Chen Q Li L Lin X Chang SI Li Y Serum anion gap on admission predicts intensive care unit mortality in patients with aortic aneurysm. Exp Ther Med. (2018) 16:1766–77. 10.3892/etm.2018.6391 30186400 PMC6122415

